# Head impacts in university-level varsity cheerleading athletes: an exploratory study

**DOI:** 10.3389/fspor.2026.1755231

**Published:** 2026-02-02

**Authors:** Emilie Croteau, Eric Wagnac, Juliette Gallant, Audrey-Anne Binet, Maée Camara, Laurie-Ann Corbin-Berrigan

**Affiliations:** 1 Department of Human Kinetics, University of Quebec in Trois-Rivieres, Trois-Rivieres, QC, Canada; 2 Département de génie mécanique, École de Technologie Supérieure, Montreal, QC, Canada; 3 Centre Intégré Universitaire de Santé et de Services Sociaux du Nord-de-l’Île-de-Montréal (CIUSSS-NIM), Montreal, QC, Canada; 4 Research Group on Musculoskeletal Disorders (GRAN), University of Quebec in Trois-Rivieres, Trois-Rivieres, QC, Canada; 5 Regroupement Intersectoriel de Recherche en Santé de L’Université du Québec (RISUQ), Montreal, QC, Canada

**Keywords:** cheerleading, concussion, female athlete, head impact, head kinematics, telemetry

## Abstract

Non-contact female dominant sports such as cheerleading are widely understudied when it comes to head kinematics and impacts. Increased knowledge could help address the current male-dominant literature and reduce sex-based disparities in research. This expanded understanding would also improve knowledge about the physiological effects of head impacts, including concussions, in women. The objective of this study was to characterize head impact (quantity and intensity) impacts in mixed biological sex cheerleading throughout the course of four regular season practices. A total of 23 university-level cheerleading athletes (17 females, 6 males) with a mean age of 21.70 ± 2.03 and 4.87 ± 4.59 years of cheerleading experience were included. A total of 89 impacts over the spend of four practices were compiled. The highest number of recorded head impacts for any athlete over the course of the study was 35, all occurring across three practice sessions; this total, when averaged over the time spent practicing, corresponds to an estimated rate of approximately 6.07 impacts to the head per hour of cheerleading activity. On an individual level, participants sustained an average of 1.93 ± 3.42 impacts per practice. The mean linear acceleration of recorded impacts was 14.72 ± 19.43 g, while the mean rotational acceleration was 804.71 ± 208.84 rad/s². Cheerleading athletes accumulate numerous impacts to the head during cheerleading practices, making them at risk of the detrimental effects of repeated head impacts.

## 1 Introduction

The practice of cheerleading is wide spread with 7.5 million athletes over 116 countries with the main pool of athletes coming from the United States of America (USA) with 3.47 million athletes in 2021 (1). According to the National Center for Catastrophic Sport Injury Research (NCCSIR), cheerleading is responsible for over half of all catastrophic injuries in female athletes (2). Meanwhile, only 28% of cheerleading injuries consult in the emergency room, underestimating greatly the true volume of injury occurring (3). Regardless of partial injury reporting, cheerleading is the sport responsible for the most average days of school missed per injury on a sample of athletes from 13 to 22 years old (4). Between 1990 and 2002, cheerleading injuries leading to a consultation at the emergency room doubled regardless multiple modifications to the rules to improve safety (5). In cheerleading, the most common causes of injury are stunts and falls, with the most common injured body parts being the head and the face (18.8%–43.3%), followed by lower extremities (11.7%–30.0%) (5–7). Concussions alone are responsible for 6.3%–31.1% of all cheerleading injuries (6, 7).

Concussions stem from a direct or indirect force to the head causing the brain to shear or collide with the skull, resulting in physical and cognitive symptoms (8). Currently, there is limited consensus on an objective tool to measure or diagnose concussions (8). There is a diversity of clinical tools based on the clinician's critical opinion and measuring subjective effects of concussion. Concussions are difficult to assess in clinic, especially when there is a time lapse between the head impact responsible for the concussion and the assessment (8). Therefore, researchers have been actively working on objective measures of concussion both in clinical and field settings. One avenue to allow for better concussion detection is to study head impact kinematics, to better understand the mechanisms linked with concussion. Indeed, it is suggested that magnitude of the impact and rotational acceleration initiated by the impact account for more than 90% of the total strain on the brain (9). However, not all impacts to the head will result in a concussion, those impacts are referred to as sub-concussive. These impacts are usually of smaller intensity, but are known to cause physiological brain changes and long-term effects, similarly to concussion (10).

Telemetric head impact sensors can help quantify the kinematics of repeated impacts to the head, including sub-concussive and concussive impacts. Previous studies have allowed to establish injury thresholds in athletes for various metrics. For instance, head impact magnitudes at 66 g, 82 g, and 98 g to 106 g are associated with a likelihood to sustain a concussion at 25%, 50%, and 80%, respectively (11, 12). Literature shows a threshold for peak rotational acceleration causing a concussion caused by rotational acceleration at 4,500 rad/s^2^ (13). Meanwhile, the HIC score, used to estimate the likelihood of moderate, serious and severe head injury, at a score of 1,000 are respectively 90%, 55% and 18% (14). Head impact sensors are mostly used in contact and helmeted sports, where sensors are worn by athletes in protective gear. Telemetric studies have widely contributed to the study of head impact in sport, but not all sports have been equally studied (15). While a substantial amount of data were measured on male American athletes, there is need for additional studies focusing on Canadian athletes, and non-contact sport athletes at risk of head impacts, such as in the case of cheerleading (16). Hence, this exploratory study aimed to characterize head impact (quantity and intensity) in mixed biological sex cheerleading during four regular season practices.

## 2 Material and methods

### 2.1 Participants

A convenience sample consisting of athletes from the mixed sex cheerleading team of the University of Québec in Trois-Rivieres (UQTR) was recruited. Inclusion criteria involved their active participation in the specified cheerleading practices during the data collection period in the 2022-23 sporting season. Participants were excluded from the study is they had an ongoing medical reason, such as active injury, preventing them from engaging in physical activity. The study was approved by the UQTR's research ethics board (CER-21-276-07.15). Participants provided written consent.

### 2.2 Procedure

Participants wore fitted headbands in which a CUE sport sensor (Athlete Intelligence, WA, USA) was inserted during a total of four consecutive regular season practices. These practices were specifically selected from the second half of the sporting season (winter semester), a period when athletes typically rehearse full competition routines rather than isolated sections. At this stage, practices included complete run-throughs of the choreography and high-risk maneuvers such as stunts and pyramids, closely replicating the conditions and intensity of actual competitions. Participants wore the headbands from the beginning to the end of the practice with the sensor positioned at the base of their heads, below the right ear, as suggested by the manufacturer. The sensor comprises two primary components: a tri-axial accelerometer and a low power inertial measurement unit (IMU), housing an additional tri-axial accelerometer and a tri-axial gyroscope (17). Recorded head impacts are sent on the Athlete intelligence's platform through a bluetooth signal to allow data tracking and data extraction for analysis. At each time-point, the following metrics were recorded to qualify the impacts: impact magnitude (peak magnitude of the resultant linear acceleration vector), peak rotational acceleration, and the HIC score (calculated using the linear acceleration at the center of mass of the head during an impact 12). No minimum threshold of data collection was established as there is limited consensus in the literature regarding an appropriate cutoff for head impact detection. Previous studies have shown that the choice of threshold can substantially influence recorded impacts, regardless of their magnitude (18, 19). To ensure transparency and avoid underestimating exposure, we included all recorded impacts regardless of their magnitude. This approach was further supported by evidence from football research using similar sensor technology, where video analysis confirmed that impacts below the commonly used threshold are still observable and may contribute to cumulative head impact burden (19).

Practices were video recorded by a research assistant, which was also present during every data collection to ensure consistency and to allow adjustments in case of material dysfunction. Start and end time of every practice were documented, as well as instrumentation inconsistencies (such as a headband falling, or early removal of headband during the practice) to account for aberrant impacts and potential loss of data. Therefore, all head impacts were observer validated by the research assistant, in accordance with recommended best practices (20).

### 2.3 Analysis

Descriptive statistics were performed for all measures, for with means and standard deviations for participants characteristics and means, standard deviations, medians, and ranges for telemetric data. Statistical analyses were performed on IBM SPSS 26.

## 3 Results

A total of 23 participants (17 females, 6 males) with a mean age of 21.70 ± 2.03 years and 4.87 ± 4.59 years of cheerleading experience were recruited (Table 1). Participants occupied the following cheerleading positions: flyers (*n* = 8), base & back spot (*n* = 3), base (*n* = 5), base & flyer (*n* = 1), gymnast (*n* = 1), and back spot (*n* = 5). Among the participants, ten (43.5%) reported having sustained a minimum of one concussion in the past with four (17.4%) of them reporting more than one concussion (Table 1).

**Table 1:** Participants’ demographic information.

Characteristics	Female	Male	Overall
	*n* = 17 (74%)	*n* = 6 (16%)	*n* = 23
Positions (*n*)	*Flyer (n* *=* *8)*		*Flyer (n* *=* *8)*
	*Base & back (n* *=* *3)*	*Base (n* *=* *1)*	*Base & back (n* *=* *3)*
	*Base (n* *=* *4)*		*Base (n* *=* *5)*
	*Base & flyer (n* *=* *1)*	*Back spot (n* *=* *5)*	*Base & flyer (n* *=* *1)*
	*Gymnast (n* *=* *1)*		*Gymnast (n* *=* *1)*
			*Back spot (n* *=* *5)*
Age (yrs) (mean ± SD)	21.35 ± 1.66	22.67 ± 2.80	21.70 ± 2.03
	*(Range:19*–*25)*	*(Range: 18*–*26)*	*(Range: 18*–*26)*
Height (cm) (mean ± SD)	159.76 ± 10.63	183.50 ± 7.97	165.96 ± 14.50
	*(Range: 125*–*173)*	*(Range: 172-194)*	*(Range: 125*–*194)*
Weight (Kg) (mean ± SD)	58.98 ± 9.05	89.39 ± 8.94	66.92 ± 16.25
	*(Range: 45*–*79)*	*(Range: 75*–*100)*	*(Range: 45*–*100)*
Years of cheerleading experience (yrs) (mean ± SD)	6.35 ± 4.42	0.67 ± 1.21	4.87 ± 4.59
	*(Range: 0*–*15)*	*(Range: 0*–*3)*	*(Range: 0*–*15)*
History of mTBI (*n*) (mean ± SD)	0.94 ± 1.43	0.50 ± 0.84	0.83 ± 1.30
	*(Range: 0*–*5)*	*(Range: 0*–*2)*	*(Range: 0*–*5)*

We collected the data over four complete and consecutive practices, which represents approximately 8 hours of data collection per participant. Practices took place in the month of January, corresponding to the mid-point of the season, where competition routines (including stunts, areal skills and tosses) are being practiced. A total of 89 head impacts were recorded and validated by the onsite observer (research assistant), corresponding to an average of 22.25 ± 11.90 impacts per practice. On average participants individually sustained 1.93 ± 3.42 impacts per practice (median: 0, range: 0–13). We recorded a mean impact magnitude of 14.72 ± 19.43 g, with a median of 10, a minimum of 1 g and maximum of 141 g. With regards to rotational acceleration a mean of 804.71 ± 208.84, a median of 780.80 rad/s^2^ (±209.0), a minimum of 420.50 rad/s^2^ and maximum of 1,534.59 rad/s^2^ were recorded. Finally, we recorded a mean HIC score of 32.86 ± 132.90, with a median of 6.96, a minimum of 1.64 and a maximum of 1,115.87.

**Figure 1:**
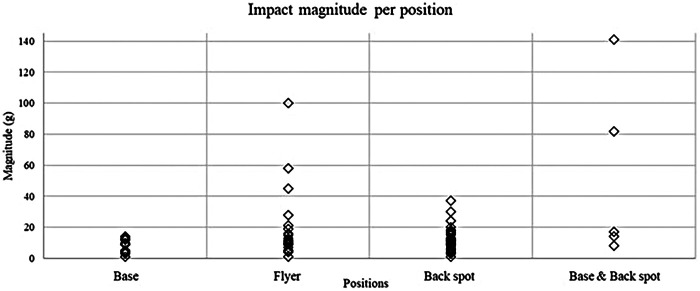
Impact magnitude per cheerleading position.

Over the course of the study, the participant with the largest amount of impact recorded was a flyer, who cumulated 35 impacts over three practices totaling 5h46 of data collection, which is equivalent to 6.1 impacts to the head per hour of cheerleading. The impact of highest magnitude was recorded by a base & back spot and was of 141 g (Figure 1). The highest rotational acceleration was recorded by a flyer at 1,534.6 rad/s^2^ and the highest HIC score was recorded by a base & back spot at a value of 1 115.9.

With regards to concussion risk thresholds, 86 (96.6%) impacts were below 66 g, one (1.1%) impact was between 66 g and 82 g, and two (2.2%) impacts were above 82 g. All impacts were below 4,500 rad/s^2^ and solely 1 (1.12%) impact with a HIC score above the 1000 threshold for brain injury was recorded. No concussion was reported during the practices in which the study took place.

## 4 Discussion

We aimed to characterize head impact (quantity and quality) in cheerleading during regular season cheerleading practices, contributing to the scarce body of literature on the topic. Our study showed a vast range of head impact magnitudes (1 g–141 g), rotational accelerations (420.50 rad/s^2^–1534.59 rad/s^2^) and HIC scores (1.64–1115.87) within the 89 head impacts measured. In Canadian varsity football, Corbin-Berrigan & al. recorded impacts to the head over multiple regular season games in a selected group of players using the CUE sport sensor, similar to this study, with the highest recorded head impact magnitude at 113 g (average: 61.6 ± 21.3 g)^16^, which is 28 g below the highest recorded impact during the four cheerleading practices included in this study. In the same study by Corbin-Berrigan & al., the highest recorded HIC score was 486.8 (average: 139.8 ± 111.7) (16) while the highest HIC score in our study was 1,115.9.

To the best of our knowledge, only one published research study has characterized head impacts in cheerleading, although the sport is highly practiced in the world and is known to produce a high volume of concussion. Allenstein et al., recorded 100 impacts to the head over 8 practices using a SIM-G sensor (21). The recorded impacts had a mean linear acceleration of 42.4 ± 15.9 g (Range: 17.7–99.4) and a mean rotational acceleration of 5600 rad/s^2^ ± 2600 (Range: 900–13300 rad/s^2^) (21). We can also draw similarities with impacts recorded in other sports where head impacts were measured with headband devices in soccer (SIM-G) (*n* = 1316), as well as in helmets in football (CUE sport sensor) (*n* = 56) (16, 22). They recorded impacts of mean linear acceleration in soccer at 43.2 ± 1.0 g, and in football at 20.08 ± 3.05 g (Max: 113) (16, 22). These findings suggest that although cheerleading is considered a non-contact sport, participants experience impacts to the head of similar or higher magnitude than football athletes, with similar experimental setup. Despite the accuracy of the CUE sport sensor being mitigated, our results allow for comparison with other studies with similar setup and highlight the occurrence of head impacts in non-contact sports such as cheerleading.

Currently, impacts to the head are understudied in women as well as in predominantly feminine sports such as cheerleading. Proper data collection and studies of sub-concussive and concussive impacts in female athletes would be highly beneficial to allow proper medical care and adequate safety measures regarding return to play in sports such as cheerleading.

### 4.1 Limitations

The biggest limitation of our study was the use of a telemetric device worn inside a headband to measure head impacts in a non-helmeted sport. Meaning that due to the nature of cheerleading, the headbands would sometime fall or get displaced, thus removing potential data collection moments during the course of the practices included in the study. Since cheerleading athletes are not used to wearing equipment, recruitment rate was not optimal, since some participants preferred not to wear headband, reducing the potential pool of data. Another limitation was our sample size; however, we were still able to obtain a strong representation of the entire team for the purposes of this study. In addition, only four practices were included. Although these sessions incorporated full routines and high-risk maneuvers, the findings primarily reflect head impact exposure during a specific training phase rather than across the entire season. Consequently, our results do not capture cumulative exposure from repeated impacts over multiple months, nor do they account for variations in training intensity earlier in the season when routines are progressively built. This limits generalizability to the overall seasonal load. Finally, our study was limited by the sensors’ accuracy. Despite these limitations, the quantity of impacts recorded through the course of this study, in cheerleading athletes, is deemed reliable and observer confirmation is definitely a strength of the proposed study (22).

## 5 Conclusions

Our study shows that cheerleading athletes are at risk of repeated head impacts, as validated by recorded impacts. Despite the exploratory nature of this study, we demonstrated that amongst cheerleaders, flyers might be at greater risk of repeated head impacts. The intensity of head impacts in cheerleading remains to be studied with more valid telemetric measures, particularly given that head-impact exposure may depend not only on an athlete's position, but also on their level of experience and technical skill. Particularly given that head-impact exposure may depend not only on an athlete's position, but also on their level of experience and technical skill. As the CUE sport sensor is deemed to be reliable, but poorly accurate. Its utilization remains pertinent to quantify the incidence of head impacts in sports, helmeted or non-helmeted, but doesn’t allow for proper characterization of the recorded impacts.

## Data Availability

The raw data supporting the conclusions of this article will be made available by the authors, upon reasonable request.
